# Exposure to ambient fine particulate matter components during pregnancy and early childhood and its association with asthma, allergies, and sensitization in school-age children

**DOI:** 10.1265/ehpm.24-00105

**Published:** 2024-07-18

**Authors:** Kazue Ojima, Yoshiko Yoda, Shin Araki, Hikari Shimadera, Narumi Tokuda, Yasuhiro Takeshima, Masayuki Shima

**Affiliations:** 1 Department of Public Health, School of Medicine, Hyogo Medical University, Nishinomiya 663-8501, Japan; 2 Hyogo Regional Center for the Japan Environment and Children’s Study, Hyogo Medical University, Nishinomiya 663-8501, Japan; 3 Graduate School of Engineering, Osaka University, Suita 565-0871, Japan; 4 Department of Pediatrics, School of Medicine, Hyogo Medical University, Nishinomiya 663-8501, Japan; 5 School of Nursing, Hyogo Medical University, Kobe 650-8530, Japan

**Keywords:** Allergy, Asthma, Birth cohort study, Fine particulate matter (PM_2.5_) component, Perinatal exposure, Sensitization

## Abstract

**Background:**

Exposure to fine particulate matter (PM_2.5_) has been associated with allergic diseases, including asthma. However, information about the effects of specific PM_2.5_ components is limited. This study aimed to investigate the relationship of exposure to chemical components of PM_2.5_ during pregnancy and early childhood with the development of asthma, allergies, and sensitization in school-age children.

**Methods:**

This study included 2,408 children in the second grade of elementary school. Questionnaire surveys of respiratory/allergic symptoms and measurements of serum total IgE and specific IgE levels to house dust mite (HDM) and animal proteins were conducted. Exposures to ambient PM_2.5_ mass, sulfate (SO_4_^2−^), nitrate (NO_3_^−^), ammonium (NH_4_^+^), elemental carbon (EC), and organic carbon (OC) of PM_2.5_ in participants’ residences from conception to age six were estimated using predictive models. Multiple logistic regression analysis was used to analyze the association of respiratory/allergic symptoms and allergen sensitization with estimated exposure concentrations, after adjustment for survey year, sex, season of birth, feeding method during infancy, presence of siblings, history of lower respiratory tract infection, use of childcare facilities, passive smoking, presence of pets, mother’s age, history of allergic diseases, smoking during pregnancy, and annual household income.

**Results:**

No significant association was found between PM_2.5_ and its component concentrations and asthma. However, wheezing significantly increased with mean NO_3_^−^ concentrations during pregnancy (odds ratio of 1.64 [95% confidence interval: 1.10, 2.47] for an interquartile range increase). Significant associations were also found between EC in the second trimester of pregnancy and PM_2.5_, NO_3_^−^, EC, and OC concentrations in early childhood. Higher PM_2.5_, SO_4_^−^, and NH_4_^+^ concentrations during the second trimester increased the risk of rhinitis. Sensitizations to HDM and animal proteins were significantly associated with exposure to components such as SO_4_^2−^ and NH_4_^+^ during pregnancy but not with postnatal exposure.

**Conclusions:**

Exposures to NO_3_^−^, EC, and OC during pregnancy and early childhood were associated with wheezing. SO_4_^2−^ and NH_4_^+^ exposures during pregnancy were associated with sensitization to HDM and animal proteins. Asthma was not associated with exposure to PM_2.5_ and its main components at any period.

**Supplementary information:**

The online version contains supplementary material available at https://doi.org/10.1265/ehpm.24-00105.

## Graphical Abstract

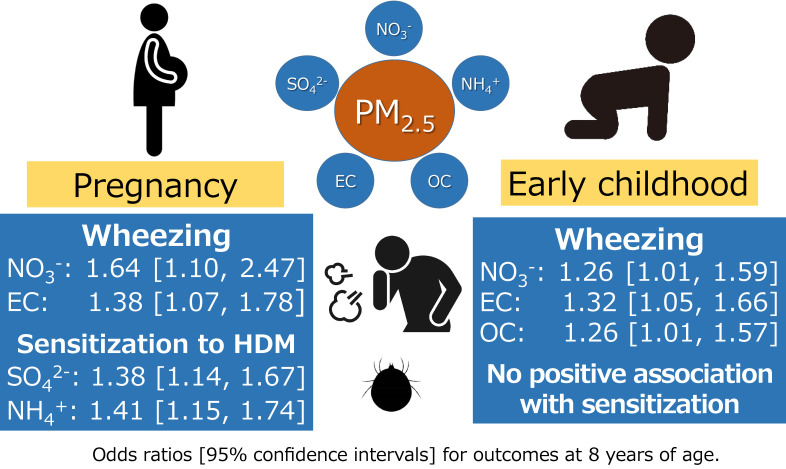

## Background

In recent years, the prevalence of allergic diseases has been on the rise among children. Both genetic [1] and environmental [2] factors are involved in this increase. Additional variables such as region, lifestyle, dietary habits, socioeconomic status, climate, and medical systems may be involved [3]. Notably, exposure to air pollutants is one of the most important environmental factors contributing to the development of allergies and asthma [4].

The influence of ambient fine particulate matter with a particle size less than 2.5 µm (PM_2.5_) has garnered increased attention [5–7]. PM_2.5_ is considered to permeate the maternal alveoli and placental barrier during the fetal period and directly affect the fetus [8]. During the fetal period, the lungs are immature and thus susceptible to environmental factors [9]. Many birth cohort studies conducted in Western countries have reported an association between exposure to air pollutants, including PM_2.5_, during the fetal period and early childhood and the onset of wheezing and asthma in childhood [5, 6, 10, 11]. A meta-analysis of these studies revealed that maternal exposure to PM_2.5_ during pregnancy was associated with wheezing and asthma in children up to three years of age [12]. Additionally, it has been reported that exposure to air pollutants, such as PM_2.5_, poses a risk of allergic rhinitis and allergen sensitization in children [13–15]. In contrast, a meta-analysis of five European birth cohort studies found no association between exposure to air pollutants, including PM_2.5_, in the prenatal and postnatal periods and childhood asthma, rhinoconjunctivitis, or allergen sensitization [16, 17]. These findings indicate the absence of a definitive conclusion regarding the association between exposure to air pollutants in the prenatal and postnatal periods and childhood allergic diseases such as asthma.

Ambient PM_2.5_ comprises carbon and various ion species [18]; however, the components that affect health have not been identified. A birth cohort study in the United States reported an association between ambient nitrate (NO_3_^−^) exposure during the fetal period and the onset of asthma in 6-year-old children [19]. Similar observations in Canada and China demonstrated that exposure to black carbon (BC), NO_3_^−^, and other components during the fetal period and early postnatal periods increased the incidence of childhood asthma [20, 21]. However, the features of PM_2.5_, including concentration, composition, and source, vary significantly depending on the region. While ambient PM_2.5_ concentration in Japan has shown improvement [22], it still exceeds the guideline level for PM_2.5_ (annual mean value of 5.0 µg/m^3^ or less) revised in 2021 by the World Health Organization (WHO). The effects of PM_2.5_ exposure during the prenatal and postnatal period on children have been reported even in the United States [19], Canada [20], and Australia [23], where levels of air pollution are lower than in Japan. However, little is known in Japan about the effects of air pollutant exposure during the fetal period, and there is little knowledge about its association with specific components.

Evaluations based on birth cohort studies are preferable for elucidating the influence of environmental factors on childhood allergic diseases such as asthma. The Japan Environmental and Children’s Study (JECS), a birth cohort study conducted by the Ministry of the Environment of Japan, is being conducted in 15 regions across the country [24]. As part of this study, we are conducting a follow-up survey of approximately 5,000 children in Amagasaki City, Hyogo Prefecture. Amagasaki is an industrial city located in western Japan and has experienced severe air pollution in the past. Although the concentrations of air pollutants, including PM_2.5_, have gradually decreased recently, the effects on asthma and allergies, which are common diseases in school-age children, have not been investigated. This study aimed to evaluate asthma and allergies among children participating in the JECS in the second grade of elementary school (8 years old) to clarify associations with individual exposure concentrations to ambient PM_2.5_ and its chemical components estimated from conception to six years of age.

## Methods

### Study design and participants

This was an Adjunct Study of the JECS. The protocol of the JECS and the baseline profiles of the participants have been described in other studies [24, 25]. The JECS is an ongoing nationwide birth cohort study that recruited approximately 100,000 pregnant women across 15 regions in Japan from January 2011 to March 2014. After registration, expecting mothers completed self-administered questionnaires twice during pregnancy: in the first and second/third trimester. Children were followed up primarily through self-administered questionnaires completed by their mothers or guardians 1 month after birth and subsequently every 6 months. The characteristics of the JECS participants were confirmed to be comparable to those of the general Japanese population [25]. The Hyogo Regional Center covers approximately 5,000 participants in Amagasaki City, Hyogo Prefecture. Amagasaki is located in an urban district in western Japan with a land area of 50.7 km^2^ and a population of approximately 458,000 as of 2023.

This study was conducted as a supplementary survey to the face-to-face examinations conducted by the JECS during the second year of elementary school for participating children. The participants, born between 2011 and 2014, underwent examinations conducted over four years from FY2019 to 2022 when the participants were in the second grade of elementary school. Examination sites were set up at public facilities in the survey region during long vacations or weekends. Children came to the sites with their guardians to undergo physical measurements, urine tests, and mental and neurological developmental assessments. This supplementary survey included a questionnaire survey regarding asthma and allergies and blood sampling.

**Fig. 1:**
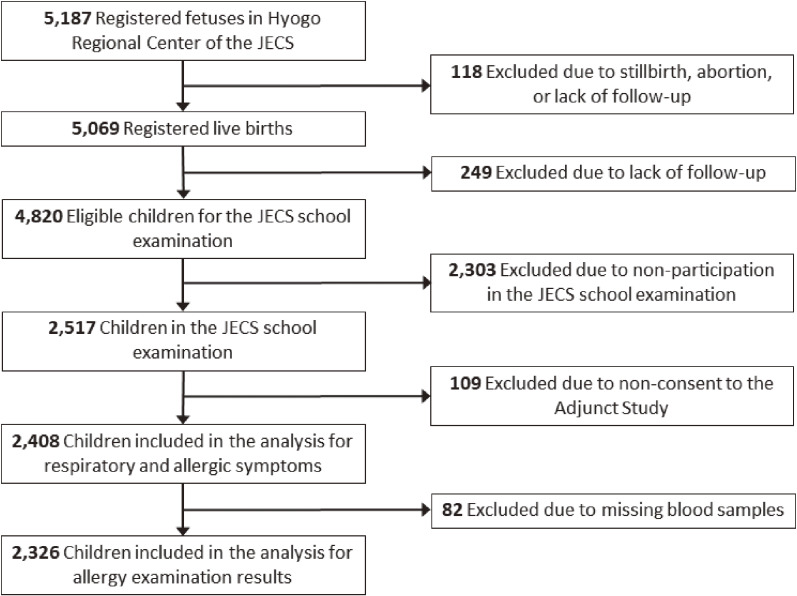
Flowchart of study participant selection. Abbreviations: JECS, Japan Environment and Children’s Study.

Detailed instructions for the supplementary survey and a guidance document for the school examination were sent via mail. Those whose written consent was obtained through written and verbal explanations given to their guardians (proxy legal guardians) when they arrived at the examination site were selected as participants. In total, 2,517 children (52.2% of the JECS registrants during this study period) participated in the JECS school examinations. Of these, 2,408 children consented to the Adjunct Study and completed a questionnaire on respiratory/allergic symptoms. Blood samples were collected from 2,326 children. Consequently, 2,408 and 2,326 children were included in the analyses for respiratory/allergic symptoms and allergy examinations, respectively (Fig. 1). This study was approved by the Hyogo Medical University ethical review committee (approval number: 3193; May 14, 2019).

### Health impact evaluation items

For assessing the respiratory/allergic symptoms of the participants, guardians completed a questionnaire based on the American Thoracic Society, Division of Lung Diseases (ATS-DLD-78-C) [26] and the International Study of Asthma and Allergies in Childhood (ISAAC) [27], widely used internationally. This aimed to evaluate the presence or absence of asthma-related symptoms, wheezing, rhinitis, and rhinoconjunctivitis.

According to the responses to the questionnaire, participants were determined to have a history of asthma based on “yes” answers to the following five questions: “Has your child ever had an attack of wheezing or whistling accompanied dyspnea?” “Has he/she ever had 2 or more such episodes?” “Has he/she been ever diagnosed with asthma by a physician?” “On that occasion, did his/her chest sound wheezy or produce a whistling sound?” and “At that time, did he/she have difficulty in breathing, accompanied by wheezing or whistling?” Wheezing was defined as two or more instances of wheezing or whistling in the chest during the past 2 years. Rhinitis was based on “yes” to the following two questions: “Has your child ever had a problem with sneezing or runny or blocked nose when he/she did not have a cold or the flu?” and “In the past 12 months, has your child had a problem with sneezing, or runny or blocked nose when he/she did not have a cold or the flu?” Rhinoconjunctivitis was defined as rhinitis with itchy-watery eyes in the past 12 months.

Additionally, 5 mL of venous blood was collected from the children, and serum total IgE and specific IgE antibody titers to house dust mite (HDM, *Dermatophagoides pteronyssinus*) and animal proteins (a mix of Cat dander, Dog dander, Guinea pig epithelium, Rat, and Mouse) were quantified using the ImmunoCAP method (Thermo Fisher Scientific, Inc., Uppsala, Sweden). The detection limits of total IgE and specific IgE were 5 IU/mL and 0.1 UA/mL, respectively. Serum total IgE of 170 IU/mL or more and specific IgE of 0.35 UA/mL or more were considered positive [13, 28].

### Exposure assessment

The concentrations of PM_2.5_ mass and main chemical component (sulfate, SO_4_^2−^; nitrate, NO_3_^−^; ammonium, NH_4_^+^; elemental carbon, EC; organic carbon, OC) during pregnancy and early childhood were estimated daily with a spatial resolution of 1 km × 1 km by daily prediction models of PM_2.5_ mass and main chemical components from 2010 to 2020, which ranged from the participants’ conception to six years of age.

Details of the model construction have been reported separately [29, 30]. In summary, PM_2.5_ and its main components were modeled using random forest, a type of machine learning, with various predictors including chemical transport model outputs, meteorological parameters, associated criteria pollutant concentrations, and traffic and land use variables. The model predicted the daily variations well, and Pearson’s correlation coefficients between the predictions and observed values at continuous monitors were 0.75–0.88 for individual components. The residence from conception until six years of age (including relocation information) for each participant was geocoded into a 1 km × 1 km grid consistent with the model prediction grid. Mean PM_2.5_ mass and main chemical component exposure concentrations were then estimated, considering the date of conception, birth, and relocation (if any) for the first (less than 14 weeks), second (14–27 weeks), and third (28 weeks and beyond) trimesters of pregnancy, as well as 0–1 year, 1–3 years, and 3–6 years after birth. Mean values for the entire pregnancy period and early childhood (0–6 years) were also calculated.

### Statistical analysis

Children who participated in this study during the four-year period from FY2019 to 2022 had the results of their respiratory/allergic symptoms and blood examination for allergies, as well as data obtained through questionnaires collected during their mother’s pregnancy and from birth to when they were four years old. The data collected by the JECS to date were utilized to analyze the association between the ambient concentration of PM_2.5_ mass and each main component estimated using the model constructed for this study.

The prevalence rates of respiratory/allergic symptoms and the results of allergy examinations were compared among fiscal years using a Pearson’s χ^2^-test. Each outcome was designated as a dependent variable, with the estimated exposure concentrations of the PM_2.5_ mass and each chemical component from conception to six years of age considered independent variables. A multiple logistic regression analysis was conducted after adjustment for the following covariates: survey year, sex, season of birth, feeding method during infancy, presence of siblings, history of lower respiratory tract infection, use of childcare facilities, passive smoking, presence of pets, mother’s age, history of allergic diseases, smoking during pregnancy, and annual household income. Missing values for covariates were supplemented using multiple imputations to reduce potential non-response bias. The 20 datasets for each imputed variable were created. Results were expressed as odds ratios (ORs) and 95% confidence intervals (CIs) of each outcome per interquartile range (IQR) increase of the estimated concentration of ambient PM_2.5_ mass and each chemical component. IBM SPSS Statistics version 27 (IBM, Armonk, NY) was used for analysis, and a p-value <0.05 was considered significant.

## Results

### Survey implementation status

During the four-year period from FY2019 to 2022, 2,408 children participated in the Adjunct Study, and blood samples were collected from 2,326 children (Fig. 1). Table 1 shows the characteristics of the participants. Though there are differences in the number of participants in each fiscal year due to the time of birth of the participants, consent rates for the participants in each fiscal year were as follows: FY2019, 52.9%; FY2020, 46.1%; FY2021, 51.8%; and FY2022, 52.1%. The consent rate in FY2020 was slightly low due to a period in which examinations could not be conducted amid the spread of Coronavirus Disease 2019 (COVID-19).

**Table 1:** Participant characteristics

	**Blood present (n = 2,326)**		**Blood absent (n = 82)**		**Overall (n = 2,408)**	
		
	**n**	**(%)**	**n**	**(%)**	**n**	**(%)**
Survey fiscal year						
FY2019	402	(17.3)	28	(34.1)	430	(17.9)
FY2020	786	(33.8)	17	(20.7)	803	(33.3)
FY2021	799	(34.4)	25	(30.5)	824	(34.2)
FY2022	339	(14.6)	12	(14.6)	351	(14.6)
Sex						
Female	1098	(47.2)	38	(46.3)	1136	(47.2)
Male	1228	(52.8)	44	(53.7)	1272	(52.8)
Season of birth						
March–May	553	(23.8)	29	(35.4)	582	(24.2)
June–August	601	(25.8)	15	(18.3)	616	(25.6)
September–November	607	(26.1)	21	(25.6)	628	(26.1)
December–February	565	(24.3)	17	(20.7)	582	(24.2)
Feeding method during infancy						
Breast milk only	812	(34.9)	26	(31.7)	838	(34.8)
Bottle-milk	163	(7.0)	5	(6.1)	168	(7.0)
Mixed feeding	1327	(57.1)	51	(62.2)	1378	(57.2)
Missing	24	(1.0)	0	(0.0)	24	(1.0)
Presence of siblings						
No	1046	(45.0)	43	(52.4)	1089	(45.2)
Yes	1256	(54.0)	39	(47.6)	1295	(53.8)
Missing	24	(1.0)	0	(0.0)	24	(1.0)
History of lower respiratory tract infection (up to two years old)						
Yes	235	(10.1)	5	(6.1)	240	(10.0)
No	2014	(86.6)	73	(89.0)	2087	(86.7)
Missing	77	(3.3)	4	(4.9)	81	(3.4)
Passive smoking (at three years old)						
Yes	378	(16.3)	10	(12.2)	388	(16.1)
No	1835	(78.9)	67	(81.7)	1902	(79.0)
Missing	113	(4.9)	5	(6.1)	118	(4.9)
Pets (three years)						
Yes	360	(15.5)	10	(12.2)	370	(15.4)
No	1872	(80.5)	67	(81.7)	1939	(80.5)
Missing	94	(4.0)	5	(6.1)	99	(4.1)
Childcare facility use (at three years old)						
Yes	1188	(51.1)	36	(43.9)	1224	(50.8)
No	995	(42.8)	39	(47.6)	1034	(42.9)
Missing	143	(6.1)	7	(8.5)	150	(6.2)
Age of mother (at time of birth)						
Mean (SD)	32.1	(4.8)	32.5	(4.8)	32.1	(4.8)
<30	728	(31.3)	21	(25.6)	749	(31.1)
≥30	1598	(68.7)	61	(74.4)	1659	(68.9)
Mother’s history of allergies						
Yes	1202	(51.7)	39	(47.6)	1241	(51.5)
No	1117	(48.0)	43	(52.4)	1160	(48.2)
Missing	7	(0.3)	0	(0.0)	7	(0.3)
Mother’s smoking history						
Yes	756	(32.5)	32	(39.0)	788	(32.7)
(smoking even during pregnancy)	62	(2.7)	4	(4.9)	66	(2.7)
No	1543	(66.3)	50	(61.0)	1593	(66.2)
Missing	27	(1.2)	0	(0.0)	27	(1.1)
Mother’s educational background						
≤12 years	622	(26.7)	22	(26.8)	644	(26.7)
≥13 years	1690	(72.7)	59	(72.0)	1749	(72.6)
Missing	14	(0.6)	1	(1.2)	15	(0.6)
Household annual income during pregnancy						
<4 million JPY	744	(32.0)	24	(29.3)	768	(31.9)
≥4 million JPY	1497	(64.4)	55	(67.1)	1552	(64.5)
Missing	85	(3.7)	3	(3.7)	88	(3.7)

Abbreviations: FY, fiscal year; SD, standard deviation; JPY, Japanese yen.

### Estimated concentrations of ambient PM_2.5_ mass and chemical components during pregnancy and early childhood

The annual trend of PM_2.5_ concentrations at a monitoring station in the study region is shown in Fig. S1. The concentration showed a slight increase in 2013 but thereafter gradually decreased. In 2020, a state of emergency for COVID-19 was declared by the Japanese government, but it had little effect on the annual trend of PM_2.5_ concentrations.

Table 2 shows the statistics of each estimated concentration of PM_2.5_ mass and main chemical components estimated for each participant during pregnancy and from birth to six years of age using the model constructed for this study. The mean (standard deviation) of the estimated PM_2.5_ concentration was 14.4 (1.2) µg/m^3^ during the entire pregnancy period and 13.1 (0.8) µg/m^3^ after birth, with the postnatal value being slightly lower. As shown in Table S1, the estimated concentrations of PM_2.5_ mass and main chemical components during pregnancy and early childhood were high for all components during pregnancy, gradually decreasing after birth.

**Table 2:** Estimated concentrations of PM2.5 mass and chemical components during pregnancy and after birth (µg/m^3^)

	**Mean**	**SD**	**Min**	**Percentiles**			**Max**	**IQR**

				**25%**	**50%**	**75%**		
Entire pregnancy (n = 2,380)								
PM_2.5_	14.4	1.2	11.0	13.5	14.3	15.2	18.2	1.6
SO_4_^2−^	4.06	0.42	2.81	3.74	4.04	4.34	5.37	0.60
NO_3_^−^	1.05	0.22	0.39	0.88	1.06	1.22	1.63	0.34
NH_4_^+^	1.75	0.17	1.24	1.62	1.76	1.87	2.29	0.25
EC	1.00	0.08	0.81	0.94	0.99	1.05	1.29	0.11
OC	3.29	0.15	2.73	3.18	3.29	3.40	3.73	0.22
First 6 years after birth (n = 2,352)								
PM_2.5_	13.1	0.8	10.7	12.6	13.1	13.6	15.9	1.0
SO_4_^2−^	3.62	0.20	3.11	3.48	3.64	3.76	4.22	0.28
NO_3_^−^	0.94	0.10	0.57	0.86	0.93	0.99	1.32	0.13
NH_4_^+^	1.55	0.09	1.28	1.49	1.56	1.62	1.86	0.13
EC	0.90	0.07	0.69	0.85	0.89	0.95	1.22	0.10
OC	3.15	0.11	2.59	3.08	3.15	3.23	3.53	0.15

Abbreviations: SD, standard deviation; IQR, interquartile range; PM_2.5_, particulate matter with a diameter of 2.5 µm or less; SO_4_^2−^, sulfate; NO_3_^−^, nitrate; NH_4_^+^, ammonium; EC, elemental carbon; OC, organic carbon.

### Respiratory/allergic symptom and allergy examination

The prevalence rates of asthma and wheezing were highest in FY2019 among both males and females and lowest in FY2022 for males and in FY2021 among females, though the difference was not significant. There were no differences in the prevalence of rhinitis and rhinoconjunctivitis between fiscal years (Table 3).

**Table 3:** Respiratory/allergic symptom and allergy examination results (by sex/fiscal year)

	**Respiratory/allergic symptom prevalence**					**Blood test for allergies**			

	**n**	**Asthma** **(%)**	**Wheezing** **(%)**	**Rhinitis** **(%)**	**Rhinoconjunctivitis** **(%)**	**n**	**Total IgE ≥ ** **170 IU/mL** **(%)**	**Sensitization ** **to HDM** **(%)**	**Sensitization to Animal proteins** **(%)**
Male									
2019	222	5.0	12.0	49.1	11.2	209	44.0	60.8	30.1
2020	422	5.0	8.6	51.2	10.5	412	49.8	67.0	36.4
2021	426	4.7	8.5	48.3	10.6	414	48.3	64.3	35.7
2022	202	4.3	6.4	53.3	12.4	193	56.0	71.5	35.9
Four years	1272	4.8	8.8	50.2	11.0	1228	49.8	65.7	35.0
p-value^†^		0.982	0.251	0.672	0.893		0.113	0.118	0.432
Female									
2019	208	5.3	8.7	33.3	6.7	193	32.1	48.2	24.5
2020	381	2.9	6.1	38.8	9.2	374	41.7	54.8	28.3
2021	398	1.5	4.5	36.2	7.3	385	40.5	52.3	29.9
2022	149	2.2	6.7	38.1	8.7	146	37.0	52.7	**17.8**
Four years	1136	2.8	6.1	36.8	8.0	1098	39.0	52.5	26.8
p-value^†^		0.075	0.252	0.591	0.672		0.127	0.523	0.024
Both sexes									
2019	430	5.1	10.4	41.5	9.0	402	**38.3**	54.7	27.4
2020	803	4.0	7.4	45.3	9.9	786	45.9	61.2	32.6
2021	824	3.2	6.6	42.5	9.0	799	44.6	58.5	33.0
2022	351	3.4	6.6	46.9	10.9	339	47.8	63.4	28.1
Four years	2408	3.8	7.5	43.9	9.6	2326	44.4	59.5	31.2
p-value^†^		0.380	0.107	0.328	0.768		0.038	0.066	0.108

^†^Pearson’s χ^2^ test for comparison among fiscal years. **Bold**, p < 0.05.

The percentage of serum total IgE ≥ 170 IU/mL was 49.8% for males and 39.0% for females, and the percentages of sensitizations to HDM and animal proteins were also higher among males than females. The percentages of elevated total IgE levels and sensitization to HDM were highest in FY2022 among males and FY2020 among females, though the difference was not significant. The percentage of sensitization to animal proteins in FY2022 was significantly lower among females (Table 3).

### Relationship of estimated concentrations of ambient PM_2.5_ mass and chemical components during pregnancy and early childhood with respiratory/allergic symptoms

**Fig. 2:**
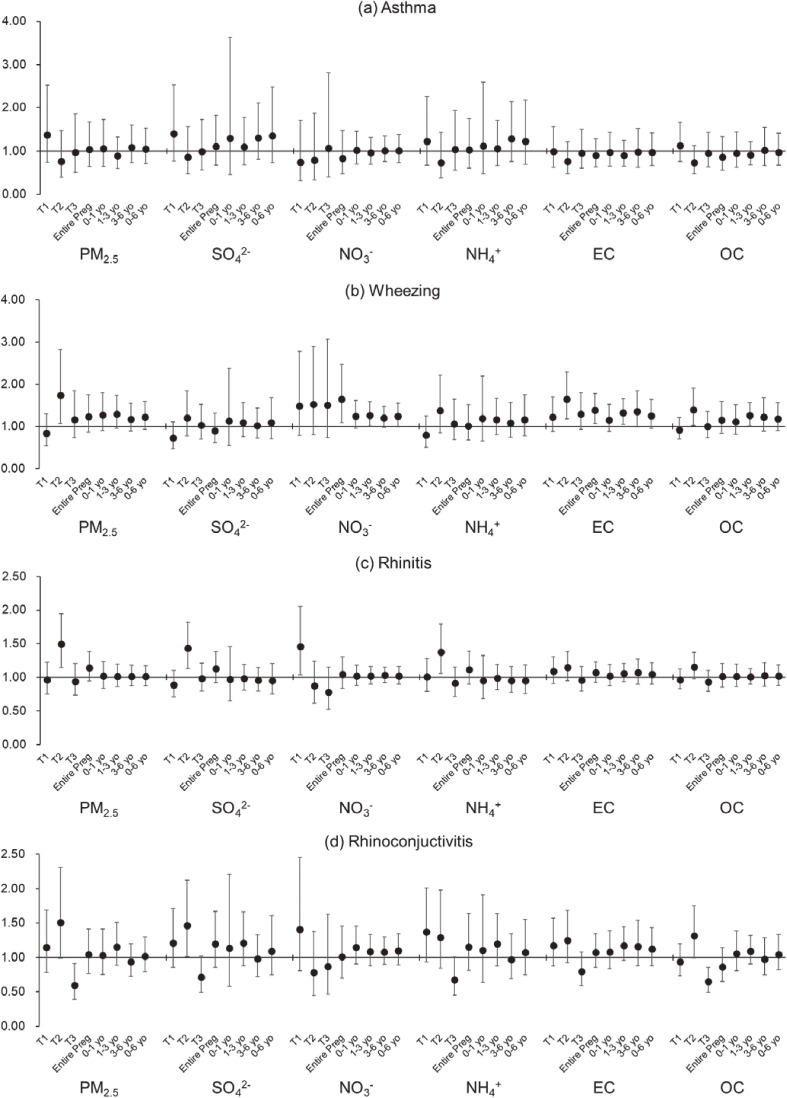
Relationship of PM_2.5_ chemical components during pregnancy and early childhood with respiratory/allergic symptoms. Data are shown as odds ratios and 95% confidence intervals for each outcome, associated with per interquartile range increase in each of PM_2.5_ mass and main chemical components, after adjustment for the covariates. Abbreviations: PM_2.5_, particulate matter with a diameter of 2.5 µm or less; SO_4_^2−^, sulfate; NO_3_^−^, nitrate; NH_4_^+^, ammonium; EC, elemental carbon; OC, organic carbon; T1, first trimester; T2, second trimester; T3, third trimester; Entire Preg, entire pregnancy; yo, years old.

Figure 2 shows the results of a multiple logistic regression analysis of the relationship of estimated concentrations of ambient PM_2.5_ mass and chemical components by period with respiratory/allergic symptoms. Detailed ORs and 95% CIs are presented in Table S2.

Asthma exhibited the largest OR per IQR increase in estimated SO_4_^2−^ concentration during the first trimester of pregnancy at 1.40 (95% CI: 0.77, 2.54); however, this was not significant. No significant associations were observed with the estimated concentrations of other components in any period. Wheezing was significantly associated with estimated NO_3_^−^ and EC concentrations throughout the pregnancy period, with ORs of 1.64 (95% CI: 1.10, 2.47) and 1.38 (95% CI: 1.07, 1.78) per IQR increase, respectively. Additionally, a significant association was found with estimated PM_2.5_, EC, and OC concentrations in the second trimester and estimated NO_3_^−^, EC, and OC concentrations in 1–3 years after birth. Rhinitis exhibited a significant association with estimated PM_2.5_ concentrations during the second trimester, with an OR of 1.50 (95% CI: 1.15, 1.95) per IQR increase. Furthermore, there was a significant association with estimated NO_3_^−^ concentrations in the first trimester and estimated SO_4_^2−^ and NH_4_^+^ concentrations in the second trimester. Rhinoconjunctivitis displayed a significant association with estimated SO_4_^2−^ concentrations during the second trimester, with an OR of 1.46 (95% CI: 1.01, 2.13) per IQR increase. Meanwhile, rhinoconjunctivitis was negatively associated with estimated PM_2.5_ and OC concentrations in the third trimester, with ORs of 0.60 (95% CI: 0.39, 0.91) and 0.65 (95% CI: 0.50, 0.86) per IQR increase, respectively.

### Relationship of estimated concentrations of ambient PM_2.5_ mass and chemical components during pregnancy and early childhood with allergy examination results

**Fig. 3:**
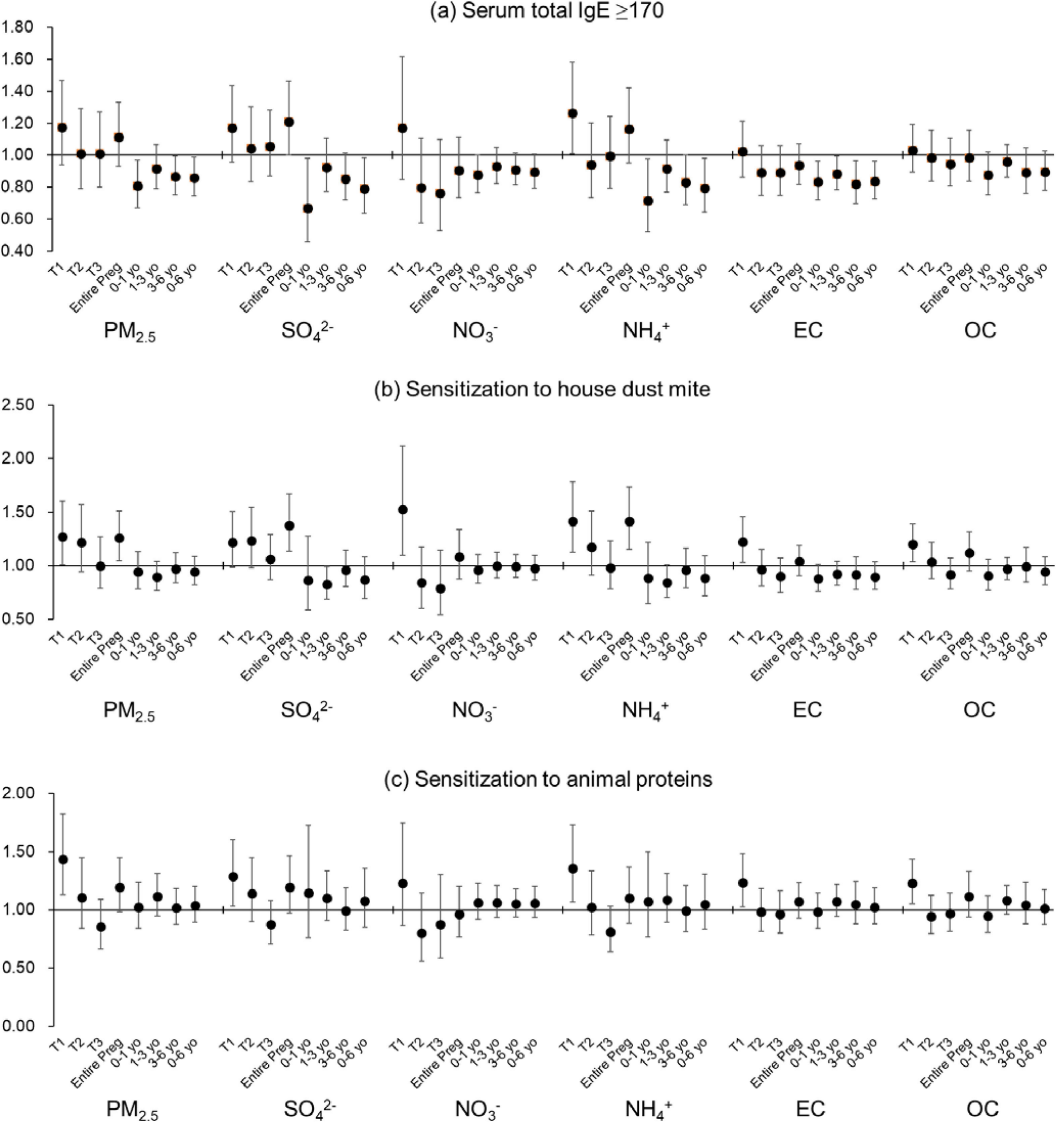
Relationship of PM_2.5_ chemical components during pregnancy and early childhood with allergy examination results. Data are shown as odds ratios and 95% confidence intervals for each outcome, associated with per interquartile range increase in each of PM_2.5_ mass and main chemical components, after adjustment for the covariates. Abbreviations: PM_2.5_, particulate matter with a diameter of 2.5 µm or less; SO_4_^2−^, sulfate; NO_3_^−^, nitrate; NH_4_^+^, ammonium; EC, elemental carbon; OC, organic carbon; T1, first trimester; T2, second trimester; T3, third trimester; Entire Preg, entire pregnancy; yo, years old.

Figure 3 shows the results of a multiple logistic regression analysis of the relationship of estimated concentrations of ambient PM_2.5_ mass and chemical components for each period with allergy examination results. Detailed ORs and 95% CIs are presented in Table S3.

High serum total IgE levels were significantly associated with estimated SO_4_^2−^ concentrations throughout pregnancy and in estimated NH_4_^+^ concentrations in the first trimester of pregnancy, with ORs of 1.21 (95% CI: 1.00, 1.46) and 1.26 (95% CI: 1.01, 1.58) per IQR increase, respectively. Conversely, higher estimated PM_2.5_, SO_4_^2−^, NH_4_^+^, and EC concentrations were found to be negatively associated at various periods after birth. Sensitization to HDM was significantly associated with estimated PM_2.5_, SO_4_^2−^, and NH_4_^+^ concentrations throughout the pregnancy period, with ORs of 1.26 (95% CI: 1.05, 1.51), 1.38 (95% CI: 1.14, 1.57), and 1.41 (95% CI: 1.15, 1.74) per IQR increase, respectively. During the first trimester, higher estimated exposures to all components other than SO_4_^2−^ were significantly associated with sensitization to HDM. However, the association with estimated SO_4_^2−^ concentration in 1–3 years after birth was significantly negative, with an OR of 0.83 (95% CI: 0.69, 0.99). Sensitization to animal proteins was significantly associated with estimated PM_2.5_ concentrations in the first trimester of pregnancy, with an OR of 1.44 (95% CI: 1.13, 1.82) per IQR increase. Significant associations were also observed with estimated SO_4_^2−^, NH_4_^+^, EC, and OC concentrations during the same period. However, no associations with concentration were observed in any other period.

## Discussion

This birth cohort study analyzed the association between the results of respiratory/allergic symptoms and blood examination for allergies in the second grade of elementary school (eight years old), and estimated concentrations of PM_2.5_ mass and main chemical component for each participant in each period during pregnancy and early childhood. Asthma did not show a significant association with estimated exposure in any period. However, estimated concentrations of PM_2.5_ during the second trimester of pregnancy and NO_3_^−^, EC, and OC throughout pregnancy and 1–3 years after birth were associated with a significant increase in wheezing. Furthermore, higher estimated concentrations of PM_2.5_, SO_4_^−^, and NH_4_^+^ during the second trimester of pregnancy were associated with a higher risk of rhinitis. For allergic status, estimated exposure to PM_2.5_ and several components during pregnancy was associated with elevated serum total IgE levels and sensitization to HDM and animal proteins. However, there was no association with postnatal exposure.

Numerous previous reports have indicated that exposure to air pollutants such as PM_2.5_ during pregnancy and early childhood was a risk factor for the onset of asthma, wheezing, rhinitis, and allergen sensitization in childhood [2, 10–15, 21]. However, a cohort study in Sweden found no association between PM_2.5_ exposure during pregnancy and one year after birth and asthma, although PM_2.5_ exposure during the first three years of life was a risk factor for asthma onset [31]. A systematic review of the association of air pollution during pregnancy with asthma and wheezing in children indicated that exposure to nitrogen dioxide (NO_2_) and sulfur dioxide (SO_2_) was a clear risk factor but that there was insufficient evidence for exposure to PM_2.5_ and BC [32]. However, some European birth cohort studies showed an association between postnatal PM_2.5_ concentration and asthma onset after the age of four years when following up to the age of 14–16 years [6]. These results show the lack of consensus on the effects of prenatal and postnatal PM_2.5_ exposure on asthma and wheezing onset in children. In the present study, there was no association between estimated exposure to PM_2.5_ and its main chemical components during pregnancy and childhood and asthma at eight years of age. However, elevated NO_3_^−^, EC, and OC exposure concentrations during pregnancy and up to three years after birth were associated with a significant increase in the prevalence of wheezing. These findings align with the results of some previous studies [11, 19, 20] conducted in countries, where air pollution levels were considerably low. Since NO_3_^−^ is generated secondarily from nitrogen oxides emitted mainly by automobiles and EC is predominantly emitted by diesel automobiles [33], it is suggested that traffic-related air pollution exerts a substantial impact.

A systematic review examining the relationship between air pollution and rhinitis indicated that children were more susceptible to air pollutants including PM_2.5_ [15, 34]. However, this association was shown to vary by country and region, with the effect being more pronounced in developing countries in Asia, where air pollution levels are high [14]. Regarding allergen sensitization, several studies reported an association between exposure to PM_2.5_ and BC and sensitization to airborne and food allergens [15, 35]. Notably, no such association was observed in a study conducted in Norway, possibly due to its low air pollution levels [36]. A meta-analysis of five European birth cohort studies did not find an association between postnatal exposure to air pollution and rhinoconjunctivitis/allergen sensitization in children aged 4–8 years [16, 17]. Of these five studies, four reported that air pollution exposure did not increase the risk of rhinoconjunctivitis even when following up until the age of 16 years [6]; however, an association with sensitization to grass pollen and cat allergen was observed [37]. In China, a study reported an association between NO_2_ exposure during pregnancy and one year after birth and allergic diseases such as hay fever and rhinitis in children aged 4–6 years, although PM_2.5_ was not specifically investigated [38]. Meanwhile, a birth cohort study in Canada found that food and inhaled allergen sensitization at one year of age was associated with traffic-related air pollution exposure one year after birth but was not associated with air pollution during pregnancy [39]. Another study demonstrated that diesel exhaust particle exposure increased airborne allergen sensitization at 2–3 years of age and was a risk factor for rhinitis onset at four years of age [40]. Additionally, it was reported that the oxidative potential of PM_2.5_ was associated with rhinitis onset but not with sensitization [41]. In this study, exposure to PM_2.5_, SO_4_^2−^, NO_3_^−^, and NH_4_^+^ during pregnancy was associated with an increased prevalence of rhinitis and rhinoconjunctivitis, although the concentrations were relatively low. Additionally, this study also revealed that exposure to PM_2.5_, SO_4_^2−^, and NH_4_^+^, which are considered to be influenced by long-range transport from the Asian continent [30], during pregnancy was associated with elevated serum total IgE levels as well as sensitization to HDM and animal proteins. Conversely, an opposite association was partially observed with postnatal exposure; therefore, this mechanism requires further investigation.

Ambient PM_2.5_ is generated from diverse natural and anthropogenic sources, with composition varying across countries and regions [18, 42, 43]. Primary components in particles, such as OC and BC, are released directly into the atmosphere from sources. In contrast, pollutants such as SO_4_^2−^, NH_4_^+^, and NO_3_^−^ are generated secondarily through chemical reactions in the atmosphere [33]. The association between the chemical components in PM_2.5_ and respiratory diseases or allergen sensitization in children has been examined [5]. A birth cohort study in the United States reported an association between fetal exposure to ambient NO_3_^−^ and asthma onset in six-year-old children [19]. Similarly, a retrospective cohort study in Canada demonstrated an association between exposure to PM_2.5_, BC, NH_4_^+^, NO_3_^−^, and organic matter during pregnancy and early childhood and asthma onset up to six years of age [20]. In China, where PM_2.5_ concentrations are significantly higher than in the United States and Canada, an association was observed between PM_2.5_ exposure during pregnancy and early childhood and asthma/wheezing onset up to six years of age, particularly combustion-derived BC, organic matter, and SO_4_^2−^ [21]. A recently reported cohort study of singleton births in Denmark revealed that PM_2.5_ exposure during pregnancy and childhood was associated with asthma onset up to 19 years of age. This study also identified associations with EC, OC, SO_4_^2−^, and NH_4_^+^ in PM_2.5_ with OC, derived mainly from biomass combustion, showing a particularly substantial influence [11]. In low- and middle-income countries in Asia, Africa, and Central/South America, the effects of exposure to household PM_2.5_ from cooking and heating on adverse perinatal outcomes and reduced lung function among children [44–46].

Many unknown aspects exist regarding the biological mechanisms by which PM contributes to the onset of asthma in children. PM_2.5_ can permeate the maternal alveoli and placental barrier during the fetal period [8]. It has also been suggested that PM may induce systemic inflammation in pregnant mothers, reducing nutrient and oxygen supply to the fetus and indirectly affecting fetal lung function [8]. After birth, environmental factors induce oxidative stress and damage in children, known to result in airway wall remodeling, initiation of inflammatory pathways and immunological effects, and enhanced respiratory sensitization to allergens [47, 48].

It has been proposed that the effects of air pollutants on rhinitis onset are mainly due to oxidative stress and inflammatory responses in human nasal epithelial cells [49–51]. Exposure to air pollutants induces allergic immune responses, including changes in serum total IgE levels in mice [52]. Air pollutants can also adhere to pollen and induce allergies in humans sensitized to pollen [53]. These studies collectively suggest that air pollution may influence allergic diseases through immune responses.

This study has some limitations. First, this study exclusively included participants covered by the Hyogo Regional Center of the JECS, implying that most participants resided in a single city. While ambient PM_2.5_ concentrations varied within the city, the differences were fewer when compared with those across Japan, rendering it insufficient for adequately detecting the influence of exposure. Second, this study focused solely on analyzing exposure to ambient PM_2.5_ and its main components; other air pollutants were not considered. Notably, NO_2_ and SO_2_ concentrations in the target region were very low. Ozone (O_3_) concentrations were relatively high with significant seasonal variations; however, they were not considered in this study due to their widespread presence and minimal concentration differences within the target region. The JECS is being implemented in 15 regions in Japan, and it is anticipated that air pollutant exposure across Japan will be evaluated and its effects clarified in the future. Third, the concentrations of PM_2.5_ mass, carbons, and main ionic components were estimated in this study; however, elemental components were not considered. Although the proportion of elemental components in PM_2.5_ is smaller than that of carbons and ionic components, associations have been observed between elemental components such as potassium and sulfur in PM_2.5_ and asthma and rhinitis [5, 6]. Future considerations should involve examining the relationship with elemental components. Fourth, outdoor PM_2.5_ exposure concentration was estimated in this study; however, indoor exposure was not considered. Given that children spend extended periods indoors, the effects of indoor air pollution must also be considered [54, 55]. The JECS involves measurements of indoor air pollutants for some participants at 1.5 and 3 years old, and these results need to be utilized for indoor air pollution evaluations. Fifth, exposure to PM_2.5_ mass and five chemical components was estimated in different periods during pregnancy and early childhood, and the association with outcomes was analyzed, raising the possibility of repeated significance testing. We did not counter the potential problem, but we believe that the results may be important per the precautionary principle. Lastly, only outcomes at eight years old were considered in this study. There are plans in the JECS to follow up on participants, and it is anticipated that the long-term effects of air pollution on allergic disease onset or allergen sensitization will be revealed.

Despite the limitations, there are several strengths in this study. First, as the participants are those from a birth cohort study, the children’s place of residence could be precisely determined daily from conception to the age of six years, allowing for an accurate evaluation of the concentrations of PM_2.5_ and the main component for each period using an exposure concentration estimation model. Second, regular follow-up surveys from pregnancy to the present day were conducted, providing sufficient information on the past medical history, living environment, and socioeconomic status of the mother and child for appropriate adjustments during analysis. Third, guardians were interviewed face-to-face when the child was eight years old, and blood samples were collected to objectively assess the state of allergen sensitization, facilitating an analysis of the relationship with PM_2.5_ exposure both before and after birth.

The prevalence rate of asthma was slightly lower and the percentage of sensitization to HDM was higher than those of the previous study conducted in this region [28], although the study methods were different. Recently, the concentrations of air pollutants, including PM_2.5_, are gradually decreasing [29, 30]. In this study, no association between asthma and exposure to PM_2.5_ and its main components may be due to low levels of air pollution in this region. However, we could observe the associations of PM_2.5_ and its main components with wheezing, rhinitis, and allergen sensitization among children. Therefore, the findings of this study can be generalized to various regions with low concentrations of air pollution.

## Conclusions

The associations were observed between estimated exposure to NO_3_^−^, EC, and OC during pregnancy and early childhood and an increased incidence of wheezing in children at eight years of age. Additionally, increased incidence of rhinitis due to PM_2.5_, SO_4_^−^, and NH_4_^+^ exposure during pregnancy was observed. However, there was no association between asthma and exposure to PM_2.5_ and its main components at any period. Regarding allergen sensitization, an association was observed between SO_4_^2−^ and NH_4_^+^ exposure during pregnancy and sensitization to HDM and animal proteins; however, no association was observed with postnatal exposure. The components identified as being associated with allergic symptoms and sensitization in this study were primarily derived from automobiles and biomass combustion. The results emphasize the importance of addressing air pollution to mitigate the risk of wheezing, asthma, and allergies in school-age children.

## Abbreviations

NH_4_^+^: ammonium
ATS-DLD: American Thoracic Society, Division of Lung Diseases
CI: confidence interval
COVID-19: Coronavirus Disease 2019
EC: elemental carbon
HDM: house dust mite
ISAAC: International Study of Asthma and Allergies in Childhood
IQR: interquartile range
JECS: Japan Environmental and Children’s Study
NO_3_^−^: nitrate
NO_2_: nitrogen dioxide
OC: organic carbon
OR: odds ratio
O_3_: ozone
PM_2.5_: particulate matter with a diameter of 2.5 µm or less
SO_4_^2−^: sulfate
SO_2_: sulfur dioxide
WHO: World Health Organization

## Supplementary information

**Additional file 1:** **Figure S1.** Annual trend of PM_2.5_ concentrations measured at a monitoring station in the study region. **Table S1.** Estimated concentrations of PM_2.5_ mass and main chemical components by period during pregnancy and after birth (µg/m^3^). **Table S2.** Associations of estimated exposures to PM_2.5_ mass and main chemical components during different periods of pregnancy and early childhood with respiratory and allergic symptoms. **Table S3.** Associations of estimated exposures to PM_2.5_ mass and main chemical components during different periods of pregnancy and early childhood with high total IgE and allergen sensitizations.

## Declarations

### Availability of data and materials

Data will be made available upon reasonable request.
