# Supplementary material for: Exposure to ambient fine particulate matter components during pregnancy and early childhood and its association with asthma, allergies, and sensitization in school-age children
Source: Environ Health Prev Med. 2024 Jul 18;29:34. doi: 10.1265/ehpm.24-00105 (PMC11273046; doi:10.1265/ehpm.24-00105)
Supplement: Supplementary file 1 — Additional file 1: Figure S1. Annual trend of PM2.5 concentrations measured at a monitoring station in the study region. Table S1. Estimated concentrations of PM2.5 mass and main chemical components by period during pregnancy and after birth (µg/m3). Table S2. Associations of estimated exposures to PM2.5 mass and main chemical components during different periods of pregnancy and early childhood with respiratory and allergic symptoms. Table S3. Associations of estimated exposures to PM2.5 mass and main chemical components during different periods of pregnancy and early childhood with high total IgE and allergen sensitizations. [file ehpm-29-034-s001.docx]

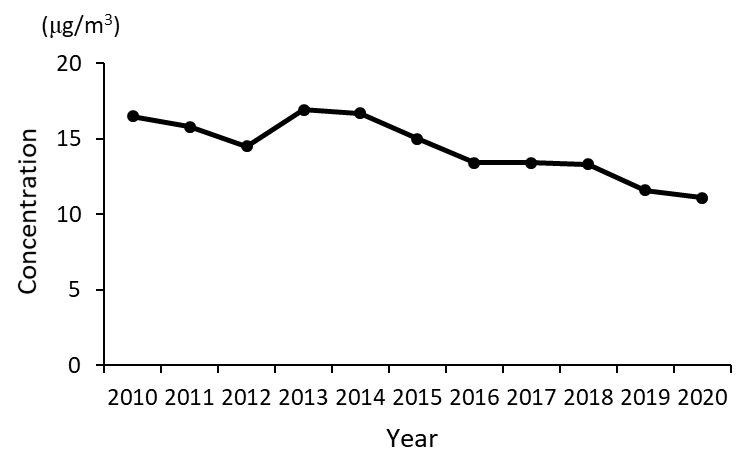


**Figure S1.** Annual trend of PM_2.5_ concentrations measured at a monitoring station in the study region.

**Table S1.** Estimated concentrations of PM_2.5_ mass and main chemical components by period during pregnancy and after birth (µg/m^3^)

|  | n | Mean | SD | Min | Percentiles | | | Max | IQR |
| --- | --- | --- | --- | --- | --- | --- | --- | --- | --- |
|  |  |  |  |  | 25% | 50% | 75% |  |  |
| PM_2. 5_ |  |  |  |  |  |  |  |  |  |
| First trimester | 2383 | 14.3 | 2.4 | 10.2 | 12.2 | 14.0 | 16.5 | 20.6 | 4.4 |
| Second trimester | 2383 | 14.4 | 2.3 | 10.2 | 12.2 | 14.2 | 16.5 | 20.3 | 4.3 |
| Third trimester | 2373 | 14.4 | 2.6 | 8.9 | 12.1 | 13.8 | 16.8 | 24.6 | 4.7 |
| Entire Pregnancy | 2380 | 14.4 | 1.2 | 11.0 | 13.5 | 14.3 | 15.2 | 18.2 | 1.6 |
| 0­–1-year-old | 2374 | 14.4 | 0.9 | 11.7 | 13.7 | 14.4 | 15.0 | 17.4 | 1.4 |
| 1–3-year-old | 2360 | 13.8 | 0.9 | 11.0 | 13.1 | 13.7 | 14.4 | 16.8 | 1.3 |
| 3–6-year-old | 2363 | 12.3 | 0.8 | 9.6 | 11.7 | 12.2 | 12.8 | 15.4 | 1.1 |
| 0–6-year-old | 2352 | 13.1 | 0.8 | 10.7 | 12.6 | 13.1 | 13.6 | 15.9 | 1.0 |
| SO_4_^2-^ |  |  |  |  |  |  |  |  |  |
| First trimester | 2383 | 4.02 | 0.88 | 2.72 | 3.24 | 3.84 | 4.74 | 6.69 | 1.49 |
| Second trimester | 2383 | 4.08 | 0.92 | 2.73 | 3.27 | 3.98 | 4.80 | 6.85 | 1.53 |
| Third trimester | 2373 | 4.10 | 1.03 | 2.46 | 3.20 | 3.89 | 4.77 | 9.12 | 1.57 |
| Entire Pregnancy | 2380 | 4.06 | 0.42 | 2.81 | 3.74 | 4.04 | 4.34 | 5.37 | 0.60 |
| 0–1-year-old | 2374 | 4.07 | 0.30 | 3.36 | 3.82 | 4.01 | 4.32 | 5.23 | 0.50 |
| 1–3-year-old | 2360 | 3.85 | 0.29 | 3.17 | 3.59 | 3.83 | 4.10 | 4.72 | 0.50 |
| 3–6-year-old | 2363 | 3.32 | 0.19 | 2.82 | 3.18 | 3.31 | 3.45 | 3.97 | 0.27 |
| 0–6-year-old | 2352 | 3.62 | 0.20 | 3.11 | 3.48 | 3.64 | 3.76 | 4.22 | 0.28 |
| NO_3_^-^ |  |  |  |  |  |  |  |  |  |
| First trimester | 2383 | 1.08 | 0.62 | 0.20 | 0.47 | 1.05 | 1.67 | 2.38 | 1.20 |
| Second trimester | 2383 | 1.05 | 0.59 | 0.20 | 0.46 | 1.01 | 1.62 | 2.37 | 1.16 |
| Third trimester | 2373 | 1.03 | 0.64 | 0.18 | 0.41 | 0.91 | 1.65 | 2.66 | 1.23 |
| Entire Pregnancy | 2380 | 1.05 | 0.22 | 0.39 | 0.88 | 1.06 | 1.22 | 1.63 | 0.34 |
| 0–1-year-old | 2374 | 1.01 | 0.11 | 0.63 | 0.93 | 1.01 | 1.08 | 1.70 | 0.15 |
| 1–3-year-old | 2360 | 0.94 | 0.11 | 0.55 | 0.86 | 0.93 | 1.01 | 1.36 | 0.15 |
| 3–6-year-old | 2363 | 0.91 | 0.09 | 0.53 | 0.84 | 0.92 | 0.97 | 1.27 | 0.13 |
| 0–6-year-old | 2352 | 0.94 | 0.10 | 0.57 | 0.86 | 0.93 | 0.99 | 1.32 | 0.13 |
| NH_4_^+^ |  |  |  |  |  |  |  |  |  |
| First trimester | 2383 | 1.75 | 0.36 | 0.98 | 1.40 | 1.80 | 2.07 | 2.51 | 0.67 |
| Second trimester | 2383 | 1.76 | 0.36 | 0.99 | 1.44 | 1.81 | 2.08 | 2.48 | 0.64 |
| Third trimester | 2373 | 1.76 | 0.40 | 0.90 | 1.40 | 1.79 | 2.10 | 3.36 | 0.71 |
| Entire Pregnancy | 2380 | 1.75 | 0.17 | 1.24 | 1.62 | 1.76 | 1.87 | 2.29 | 0.25 |
| 0–1-year-old | 2374 | 1.75 | 0.12 | 1.39 | 1.65 | 1.73 | 1.85 | 2.16 | 0.20 |
| 1–3-year-old | 2360 | 1.65 | 0.12 | 1.30 | 1.55 | 1.65 | 1.75 | 2.03 | 0.20 |
| 3–6-year-old | 2363 | 1.42 | 0.09 | 1.17 | 1.36 | 1.42 | 1.49 | 1.75 | 0.13 |
| 0–6-year-old | 2352 | 1.55 | 0.09 | 1.28 | 1.49 | 1.56 | 1.62 | 1.86 | 0.13 |
| EC |  |  |  |  |  |  |  |  |  |
| First trimester | 2383 | 1.00 | 0.14 | 0.68 | 0.90 | 1.01 | 1.10 | 1.38 | 0.20 |
| Second trimester | 2383 | 1.00 | 0.13 | 0.68 | 0.91 | 1.00 | 1.09 | 1.47 | 0.18 |
| Third trimester | 2373 | 0.99 | 0.15 | 0.60 | 0.90 | 1.00 | 1.09 | 1.54 | 0.20 |
| Entire Pregnancy | 2380 | 1.00 | 0.08 | 0.81 | 0.94 | 0.99 | 1.05 | 1.29 | 0.11 |
| 0–1-year-old | 2374 | 0.98 | 0.07 | 0.73 | 0.93 | 0.98 | 1.03 | 1.28 | 0.10 |
| 1–3-year-old | 2360 | 0.96 | 0.07 | 0.71 | 0.91 | 0.95 | 1.00 | 1.27 | 0.10 |
| 3–6-year-old | 2363 | 0.83 | 0.09 | 0.60 | 0.77 | 0.83 | 0.89 | 1.19 | 0.13 |
| 0–6-year-old | 2352 | 0.90 | 0.07 | 0.69 | 0.85 | 0.89 | 0.95 | 1.22 | 0.10 |
| OC |  |  |  |  |  |  |  |  |  |
| First trimester | 2383 | 3.29 | 0.31 | 2.46 | 3.11 | 3.31 | 3.51 | 4.03 | 0.40 |
| Second trimester | 2383 | 3.30 | 0.30 | 2.55 | 3.11 | 3.32 | 3.52 | 4.02 | 0.41 |
| Third trimester | 2373 | 3.29 | 0.34 | 2.35 | 3.08 | 3.33 | 3.54 | 4.37 | 0.46 |
| Entire Pregnancy | 2380 | 3.29 | 0.15 | 2.73 | 3.18 | 3.29 | 3.40 | 3.73 | 0.22 |
| 0–1-year-old | 2374 | 3.27 | 0.13 | 2.58 | 3.18 | 3.27 | 3.36 | 3.69 | 0.18 |
| 1–3-year-old | 2360 | 3.23 | 0.11 | 2.59 | 3.16 | 3.23 | 3.30 | 3.61 | 0.14 |
| 3–6-year-old | 2363 | 3.05 | 0.14 | 2.42 | 2.96 | 3.05 | 3.15 | 3.50 | 0.19 |
| 0–6-year-old | 2352 | 3.15 | 0.11 | 2.59 | 3.08 | 3.15 | 3.23 | 3.53 | 0.15 |

Abbreviations: SD, standard deviation; IQR, interquartile range; PM_2.5_, particulate matter with a diameter of 2.5 μm or less; SO_4_^2-^, sulfate; NO_3_^-^, nitrate; NH_4_^+^, ammonium; EC, elemental carbon; OC, organic carbon.

**Table S2.** Associations of estimated exposures to PM_2.5_ mass and main chemical components during different periods of pregnancy and early childhood with respiratory and allergic symptoms.

|  | Asthma | | Wheezing | | Rhinitis | | Rhinoconjuctivitis | |
| --- | --- | --- | --- | --- | --- | --- | --- | --- |
|  | OR | 95%CI | OR | 95%CI | OR | 95%CI | OR | 95%CI |
| PM_2. 5_ |  |  |  |  |  |  |  |  |
| First trimester | 1.37 | (0.74, 2.53) | 0.84 | (0.54, 1.29) | 0.96 | (0.76, 1.22) | 1.15 | (0.78, 1.69) |
| Second trimester | 0.76 | (0.40, 1.47) | **1.74** | **(1.07, 2.82)** | **1.50** | **(1.15, 1.95)** | 1.51 | (0.99, 2.31) |
| Third trimester | 0.97 | (0.50, 1.86) | 1.17 | (0.74, 1.85) | 0.94 | (0.73, 1.21) | **0.60** | **(0.39, 0.91)** |
| Entire Pregnancy | 1.04 | (0.65, 1.67) | 1.23 | (0.87, 1.75) | 1.14 | (0.94, 1.38) | 1.04 | (0.76, 1.42) |
| 0­–1-year-old | 1.05 | (0.64, 1.73) | 1.27 | (0.90, 1.80) | 1.02 | (0.84, 1.24) | 1.03 | (0.75, 1.41) |
| 1–3-year-old | 0.88 | (0.59, 1.32) | 1.29 | (0.96, 1.74) | 1.02 | (0.87, 1.20) | 1.16 | (0.89, 1.51) |
| 3–6-year-old | 1.08 | (0.73, 1.59) | 1.18 | (0.89, 1.55) | 1.01 | (0.87, 1.18) | 0.93 | (0.73, 1.20) |
| 0–6-year-old | 1.05 | (0.71, 1.53) | 1.22 | (0.93, 1.59) | 1.01 | (0.87, 1.18) | 1.01 | (0.79, 1.30) |
| SO_4_^2-^ |  |  |  |  |  |  |  |  |
| First trimester | 1.40 | (0.77, 2.54) | 0.73 | (0.48, 1.11) | 0.89 | (0.71, 1.11) | 1.21 | (0.85, 1.71) |
| Second trimester | 0.86 | (0.48, 1.55) | 1.20 | (0.78, 1.85) | **1.43** | **(1.13, 1.82)** | **1.46** | **(1.01, 2.13)** |
| Third trimester | 0.99 | (0.57, 1.73) | 1.03 | (0.70, 1.52) | 0.98 | (0.80, 1.21) | 0.71 | (0.50, 1.01) |
| Entire Pregnancy | 1.10 | (0.67, 1.82) | 0.90 | (0.62, 1.32) | 1.13 | (0.92, 1.38) | 1.20 | (0.86, 1.67) |
| 0–1-year-old | 1.29 | (0.46, 3.63) | 1.14 | (0.54, 2.38) | 0.97 | (0.65, 1.46) | 1.14 | (0.58, 2.21) |
| 1–3-year-old | 1.10 | (0.68, 1.78) | 1.09 | (0.76, 1.57) | 0.98 | (0.81, 1.19) | 1.21 | (0.88, 1.67) |
| 3–6-year-old | 1.30 | (0.81, 2.11) | 1.02 | (0.73, 1.44) | 0.96 | (0.80, 1.15) | 0.98 | (0.72, 1.33) |
| 0–6-year-old | 1.35 | (0.73, 2.49) | 1.09 | (0.71, 1.68) | 0.95 | (0.76, 1.20) | 1.09 | (0.74, 1.61) |
| NO_3_^-^ |  |  |  |  |  |  |  |  |
| First trimester | 0.74 | (0.32, 1.70) | 1.48 | (0.79, 2.78) | **1.46** | **(1.04, 2.06)** | 1.41 | (0.81, 2.45) |
| Second trimester | 0.79 | (0.33, 1.88) | 1.53 | (0.81, 2.89) | 0.87 | (0.62, 1.24) | 0.78 | (0.44, 1.38) |
| Third trimester | 1.07 | (0.40, 2.81) | 1.50 | (0.73, 3.07) | 0.78 | (0.53, 1.15) | 0.87 | (0.46, 1.62) |
| Entire Pregnancy | 0.83 | (0.47, 1.47) | **1.64** | **(1.10, 2.47)** | 1.04 | (0.84, 1.31) | 1.01 | (0.70, 1.46) |
| 0–1-year-old | 1.01 | (0.70, 1.46) | 1.25 | (0.96, 1.62) | 1.02 | (0.88, 1.18) | 1.15 | (0.91, 1.46) |
| 1–3-year-old | 0.95 | (0.69, 1.31) | **1.26** | **(1.01, 1.59)** | 1.02 | (0.90, 1.16) | 1.09 | (0.88, 1.34) |
| 3–6-year-old | 1.01 | (0.76, 1.34) | 1.21 | (0.98, 1.48) | 1.03 | (0.92, 1.16) | 1.08 | (0.90, 1.30) |
| 0–6-year-old | 1.00 | (0.73, 1.38) | 1.24 | (0.99, 1.56) | 1.02 | (0.90, 1.16) | 1.10 | (0.89, 1.35) |
| NH_4_^+^ |  |  |  |  |  |  |  |  |
| First trimester | 1.23 | (0.67, 2.25) | 0.80 | (0.51, 1.25) | 1.01 | (0.79, 1.28) | 1.37 | (0.94, 2.01) |
| Second trimester | 0.73 | (0.37, 1.43) | 1.37 | (0.85, 2.22) | **1.38** | **(1.06, 1.79)** | 1.29 | (0.84, 1.98) |
| Third trimester | 1.03 | (0.55, 1.93) | 1.06 | (0.69, 1.65) | 0.91 | (0.72, 1.15) | 0.67 | (0.45, 1.00) |
| Entire Pregnancy | 1.02 | (0.60, 1.75) | 1.01 | (0.68, 1.51) | 1.12 | (0.90, 1.39) | 1.15 | (0.81, 1.64) |
| 0–1-year-old | 1.12 | (0.48, 2.60) | 1.20 | (0.65, 2.19) | 0.95 | (0.68, 1.33) | 1.11 | (0.64, 1.91) |
| 1–3-year-old | 1.06 | (0.66, 1.70) | 1.16 | (0.81, 1.67) | 0.99 | (0.82, 1.20) | 1.20 | (0.87, 1.64) |
| 3–6-year-old | 1.28 | (0.76, 2.14) | 1.08 | (0.75, 1.56) | 0.95 | (0.78, 1.16) | 0.97 | (0.70, 1.35) |
| 0–6-year-old | 1.22 | (0.69, 2.18) | 1.17 | (0.78, 1.75) | 0.95 | (0.76, 1.19) | 1.07 | (0.74, 1.55) |
| EC |  |  |  |  |  |  |  |  |
| First trimester | 0.98 | (0.62, 1.56) | 1.22 | (0.88, 1.70) | 1.09 | (0.91, 1.31) | 1.17 | (0.87, 1.57) |
| Second trimester | 0.76 | (0.48, 1.21) | **1.64** | **(1.18, 2.29)** | 1.15 | (0.95, 1.38) | 1.25 | (0.92, 1.68) |
| Third trimester | 0.95 | (0.60, 1.50) | 1.29 | (0.93, 1.80) | 0.96 | (0.80, 1.16) | 0.80 | (0.59, 1.08) |
| Entire Pregnancy | 0.90 | (0.63, 1.28) | **1.38** | **(1.07, 1.78)** | 1.07 | (0.93, 1.23) | 1.07 | (0.86, 1.35) |
| 0–1-year-old | 0.97 | (0.65, 1.43) | 1.16 | (0.88, 1.53) | 1.02 | (0.88, 1.19) | 1.08 | (0.84, 1.39) |
| 1–3-year-old | 0.90 | (0.65, 1.24) | **1.32** | **(1.05, 1.66)** | 1.06 | (0.93, 1.21) | 1.17 | (0.95, 1.44) |
| 3–6-year-old | 0.97 | (0.62, 1.52) | 1.36 | (0.99, 1.85) | 1.07 | (0.90, 1.27) | 1.16 | (0.88, 1.54) |
| 0–6-year-old | 0.97 | (0.66, 1.42) | 1.25 | (0.96, 1.64) | 1.05 | (0.90, 1.22) | 1.12 | (0.88, 1.43) |
| OC |  |  |  |  |  |  |  |  |
| First trimester | 1.13 | (0.76, 1.66) | 0.92 | (0.70, 1.21) | 0.97 | (0.83, 1.13) | 0.94 | (0.73, 1.20) |
| Second trimester | 0.73 | (0.48, 1.12) | **1.40** | **(1.02, 1.91)** | 1.15 | (0.97, 1.37) | 1.32 | (0.99, 1.75) |
| Third trimester | 0.95 | (0.63, 1.43) | 1.00 | (0.74, 1.36) | 0.93 | (0.79, 1.10) | **0.65** | **(0.50, 0.86)** |
| Entire Pregnancy | 0.86 | (0.55, 1.33) | 1.16 | (0.84, 1.60) | 1.01 | (0.85, 1.20) | 0.86 | (0.65, 1.14) |
| 0–1-year-old | 0.95 | (0.62, 1.44) | 1.11 | (0.82, 1.51) | 1.02 | (0.86, 1.20) | 1.05 | (0.80, 1.38) |
| 1–3-year-old | 0.91 | (0.68, 1.21) | **1.26** | **(1.01, 1.57)** | 1.01 | (0.90, 1.13) | 1.09 | (0.90, 1.32) |
| 3–6-year-old | 1.01 | (0.67, 1.55) | 1.22 | (0.89, 1.68) | 1.03 | (0.87, 1.22) | 0.97 | (0.74, 1.28) |
| 0–6-year-old | 0.97 | (0.67, 1.41) | 1.19 | (0.90, 1.56) | 1.02 | (0.88, 1.18) | 1.04 | (0.82, 1.33) |

Abbreviations: OR, odds ratio; CI, confidence interval; IQR, interquartile range; PM_2.5_, particulate matter with a diameter of 2.5 μm or less; SO_4_^2-^, sulfate; NO_3_^-^, nitrate; NH_4_^+^, ammonium; EC, elemental carbon; OC, organic carbon.

Data are shown as odds ratios and 95% confidence intervals for each outcome, associated with per interquartile range increase in each of PM_2.5_ mass and main chemical components, after adjustment for the covariates. **Bold**, p<0.05.

**Table S3.** Associations of estimated exposures to PM_2.5_ mass and main chemical components during different periods of pregnancy and early childhood with high total IgE and allergen sensitizations.

|  | Serum total IgE ≥170 | | Sensitization to house dust mite | | Sensitization to animal proteins | |
| --- | --- | --- | --- | --- | --- | --- |
|  | OR | (95%CI) | OR | (95%CI) | OR | (95%CI) |
| PM_2. 5_ |  |  |  |  |  |  |
| First trimester | 1.17 | (0.94, 1.47) | **1.27** | **(1.01, 1.60)** | **1.44** | **(1.13, 1.82)** |
| Second trimester | 1.01 | (0.79, 1.29) | 1.22 | (0.94, 1.57) | 1.10 | (0.84, 1.45) |
| Third trimester | 1.01 | (0.80, 1.27) | 1.00 | (0.79, 1.27) | 0.85 | (0.67, 1.09) |
| Entire Pregnancy | 1.11 | (0.93, 1.33) | **1.26** | **(1.05, 1.51)** | 1.19 | (0.98, 1.45) |
| 0­–1-year-old | **0.81** | **(0.67, 0.97)** | 0.94 | (0.78, 1.13) | 1.02 | (0.84, 1.24) |
| 1–3-year-old | 0.92 | (0.79, 1.07) | 0.89 | (0.77, 1.04) | 1.12 | (0.95, 1.31) |
| 3–6-year-old | 0.86 | (0.75, 1.00) | 0.97 | (0.84, 1.12) | 1.02 | (0.87, 1.18) |
| 0–6-year-old | **0.86** | **(0.74, 0.99)** | 0.95 | (0.82, 1.09) | 1.04 | (0.89, 1.21) |
| SO_4_^2-^ |  |  |  |  |  |  |
| First trimester | 1.17 | (0.95, 1.44) | 1.22 | (0.99, 1.50) | **1.29** | **(1.03, 1.60)** |
| Second trimester | 1.04 | (0.83, 1.30) | 1.23 | (0.98, 1.55) | 1.14 | (0.90, 1.45) |
| Third trimester | 1.05 | (0.87, 1.28) | 1.06 | (0.87, 1.29) | 0.87 | (0.71, 1.08) |
| Entire Pregnancy | **1.21** | **(1.00, 1.46)** | **1.38** | **(1.14, 1.67)** | 1.19 | (0.97, 1.47) |
| 0–1-year-old | **0.67** | **(0.45, 0.98)** | 0.87 | (0.59, 1.28) | 1.15 | (0.76, 1.73) |
| 1–3-year-old | 0.92 | (0.77, 1.11) | **0.83** | **(0.69, 0.99)** | 1.10 | (0.91, 1.34) |
| 3–6-year-old | 0.85 | (0.72, 1.01) | 0.96 | (0.81, 1.14) | 0.99 | (0.82, 1.19) |
| 0–6-year-old | **0.79** | **(0.64, 0.99)** | 0.87 | (0.70, 1.08) | 1.07 | (0.85, 1.36) |
| NO_3_^-^ |  |  |  |  |  |  |
| First trimester | 1.17 | (0.85, 1.62) | **1.53** | **(1.10, 2.12)** | 1.23 | (0.86, 1.74) |
| Second trimester | 0.80 | (0.57, 1.11) | 0.84 | (0.60, 1.18) | 0.80 | (0.56, 1.14) |
| Third trimester | 0.76 | (0.53, 1.10) | 0.79 | (0.55, 1.15) | 0.88 | (0.59, 1.30) |
| Entire Pregnancy | 0.90 | (0.73, 1.11) | 1.08 | (0.88, 1.34) | 0.96 | (0.77, 1.20) |
| 0–1-year-old | 0.88 | (0.77, 1.00) | 0.96 | (0.84, 1.10) | 1.06 | (0.92, 1.23) |
| 1–3-year-old | 0.93 | (0.82, 1.05) | 1.00 | (0.88, 1.13) | 1.06 | (0.94, 1.21) |
| 3–6-year-old | 0.91 | (0.82, 1.01) | 0.99 | (0.89, 1.10) | 1.05 | (0.94, 1.18) |
| 0–6-year-old | 0.89 | (0.79, 1.00) | 0.98 | (0.87, 1.10) | 1.06 | (0.93, 1.20) |
| NH_4_^+^ |  |  |  |  |  |  |
| First trimester | **1.26** | **(1.01, 1.58)** | **1.41** | **(1.12, 1.78)** | **1.36** | **(1.07, 1.73)** |
| Second trimester | 0.94 | (0.73, 1.20) | 1.18 | (0.91, 1.51) | 1.02 | (0.79, 1.34) |
| Third trimester | 0.99 | (0.79, 1.24) | 0.98 | (0.78, 1.23) | 0.81 | (0.64, 1.03) |
| Entire Pregnancy | 1.16 | (0.95, 1.42) | **1.41** | **(1.15, 1.74)** | 1.10 | (0.89, 1.37) |
| 0–1-year-old | **0.71** | **(0.52, 0.98)** | 0.89 | (0.64, 1.22) | 1.07 | (0.77, 1.50) |
| 1–3-year-old | 0.92 | (0.77, 1.09) | 0.84 | (0.70, 1.01) | 1.08 | (0.90, 1.31) |
| 3–6-year-old | 0.83 | (0.69, 1.00) | 0.96 | (0.80, 1.16) | 0.99 | (0.81, 1.21) |
| 0–6-year-old | **0.79** | **(0.64, 0.98)** | 0.89 | (0.72, 1.09) | 1.05 | (0.84, 1.31) |
| EC |  |  |  |  |  |  |
| First trimester | 1.02 | (0.86, 1.21) | **1.22** | **(1.03, 1.46)** | **1.23** | **(1.03, 1.48)** |
| Second trimester | 0.89 | (0.75, 1.06) | 0.97 | (0.81, 1.15) | 0.98 | (0.81, 1.18) |
| Third trimester | 0.89 | (0.75, 1.06) | 0.90 | (0.75, 1.07) | 0.97 | (0.80, 1.16) |
| Entire Pregnancy | 0.94 | (0.82, 1.07) | 1.04 | (0.91, 1.19) | 1.07 | (0.93, 1.23) |
| 0–1-year-old | **0.83** | **(0.72, 0.96)** | 0.88 | (0.76, 1.02) | 0.98 | (0.84, 1.14) |
| 1–3-year-old | 0.88 | (0.78, 1.00) | 0.92 | (0.82, 1.04) | 1.07 | (0.94, 1.22) |
| 3–6-year-old | **0.82** | **(0.70, 0.96)** | 0.92 | (0.78, 1.08) | 1.05 | (0.88, 1.24) |
| 0–6-year-old | **0.84** | **(0.73, 0.96)** | 0.90 | (0.78, 1.03) | 1.02 | (0.88, 1.19) |
| OC |  |  |  |  |  |  |
| First trimester | 1.03 | (0.89, 1.19) | **1.20** | **(1.04, 1.39)** | **1.23** | **(1.05, 1.43)** |
| Second trimester | 0.98 | (0.84, 1.15) | 1.03 | (0.88, 1.22) | 0.95 | (0.80, 1.12) |
| Third trimester | 0.95 | (0.81, 1.11) | 0.92 | (0.78, 1.07) | 0.97 | (0.82, 1.14) |
| Entire Pregnancy | 0.98 | (0.84, 1.15) | 1.12 | (0.95, 1.32) | 1.12 | (0.94, 1.33) |
| 0–1-year-old | 0.87 | (0.75, 1.02) | 0.91 | (0.77, 1.06) | 0.95 | (0.80, 1.12) |
| 1–3-year-old | 0.96 | (0.86, 1.07) | 0.97 | (0.87, 1.08) | 1.08 | (0.96, 1.21) |
| 3–6-year-old | 0.89 | (0.76, 1.04) | 0.99 | (0.85, 1.17) | 1.04 | (0.88, 1.24) |
| 0–6-year-old | 0.90 | (0.78, 1.03) | 0.94 | (0.82, 1.08) | 1.01 | (0.87, 1.17) |

Abbreviations: OR, odds ratio; CI, confidence interval; IQR, interquartile range; PM_2.5_, particulate matter with a diameter of 2.5 μm or less; SO_4_^2-^, sulfate; NO_3_^-^, nitrate; NH_4_^+^, ammonium; EC, elemental carbon; OC, organic carbon.

Data are shown as odds ratios and 95% confidence intervals for each outcome, associated with per interquartile range increase in each of PM_2.5_ mass and main chemical components, after adjustment for the covariates. **Bold**, p<0.05.
